# Transcriptomic analysis of differentially expressed genes in the oviduct of *Rhacophorus omeimontis* provides insights into foam nest construction

**DOI:** 10.1186/s12864-019-5931-7

**Published:** 2019-07-08

**Authors:** Wei Zhang, Li Huang, Jun Li, Yinghua Li, Shichao Wei, Ling Cai, Hua Wu

**Affiliations:** 1 Institute of Evolution and Ecology, International Research Centre of Ecology and Environment, College of Life Sciences, Central China Normal University, Wuhan, 430079 China; 2 College of Life Science, Zhengzhou Normal University, Zhengzhou, 450044 China; 3 College of Life Science and Agronomy, Zhoukou Normal University, Zhoukou, 466000 China

**Keywords:** Transcriptome, Foam nest, *Rhacophorus omeimontis*, Oviduct, Lectin, Immune defense

## Abstract

**Background:**

The production of foam nests is one of the strategies that has evolved to allow some anuran species to protect their eggs and larvae. Despite considerable knowledge of the biochemical components of and construction behavior leading to anuran foam nests, little is known about the molecular basis of foam nest construction. *Rhacophorus omeimontis* presents an arboreal foam-nesting strategy during the breeding season. To better understand the molecular mechanism of foam nest production, transcriptome sequencing was performed using the oviduct of female *R. omeimontis* during the period when foam nest production began and the period when foam nest production was finished.

**Results:**

The transcriptomes of six oviduct samples of *R. omeimontis* were obtained using Illumina sequencing. A total of 84,917 unigenes were obtained, and 433 genes (270 upregulated and 163 downregulated) were differentially expressed between the two periods. These differentially expressed genes (DEGs) were mainly enriched in extracellular space and extracellular region based on Gene Ontology (GO) enrichment analysis and in the pathways of two-component system, cell adhesion molecules, steroid hormone biosynthesis and neuroactive ligand-receptor interaction based on Kyoto Encyclopedia of Genes and Genomes (KEGG) enrichment analysis. Specifically, genes encoding lectins, surfactant proteins and immunity components were highly expressed when the foam nest construction began, indicating that the constituents of foam nests in *R. omeimontis* were likely a mixture of surfactant, lectins and immune defense proteins. During the period when foam nest production was finished, genes related to lipid metabolism, steroid hormone and immune defense were highly expressed, indicating their important roles in regulating the process of foam nesting.

**Conclusions:**

Our study provides a rich list of potential genes involved in the production of foam nests in *R. omeimontis*. These results provide insights into the molecular mechanisms underlying the process of foam nest construction and will facilitate further studies of *R. omeimontis*.

**Electronic supplementary material:**

The online version of this article (10.1186/s12864-019-5931-7) contains supplementary material, which is available to authorized users.

## Background

Foam nest production is a breeding strategy that has evolved in some homopteran bugs, tunicates, fishes and anuran amphibians to protect their eggs and larvae from environmental challenges [1–4]. In anuran amphibians, foam nest construction is associated with adaptation to terrestrial life, and 10 out of the 41 different reproductive modes found in anurans involve foam nest construction [5–7]. Depending on the anuran species, the foam nests are produced in underground burrows, float on the water surface, or are even suspended from vegetation [4, 8–10]. Several functions have been identified for the production of anuran foam nests, such as protecting eggs and embryos against predators, desiccation, microbial degradation and thermal damage, as well as supplying oxygen and serving as a food source for tadpoles [2, 8, 11–13].

The production of foam nests has evolved independently multiple times in several lineages of anurans; thus, anurans are a particularly interesting case for which to study foam nests [6, 14]. Recent studies on anuran foam nests have mostly focused on their structure and biochemical composition and the construction behavior leading to them [4, 13, 15–17]. Well-studied foam-nesting species include frogs from the genus *Leptodactylus*, especially the túngara frog [4, 10, 12, 14, 15]. Hissa et al. [16] and Fleming et al. [17] reported that the foam nest components of *Leptodactylus* frogs were a mixture of proteins termed ranaspumins, with surfactant activity, cystatin activity and lectin composition. The foam components were mainly secreted from the posterior convolutions of the oviducts in mature female foam-nesting frogs [18, 19].

Despite considerable knowledge of the biochemical components of and construction behavior leading to anuran foam nests, little is known about the molecular basis of and metabolic pathways involved in the process of foam nest production. In addition, published studies on anuran foam nests typically concern the water-based foams produced by members of Leptodactylidae, and whether the arboreal foam nests of anuran species have similar components remains unclear [10, 20].

**Fig. 1:**
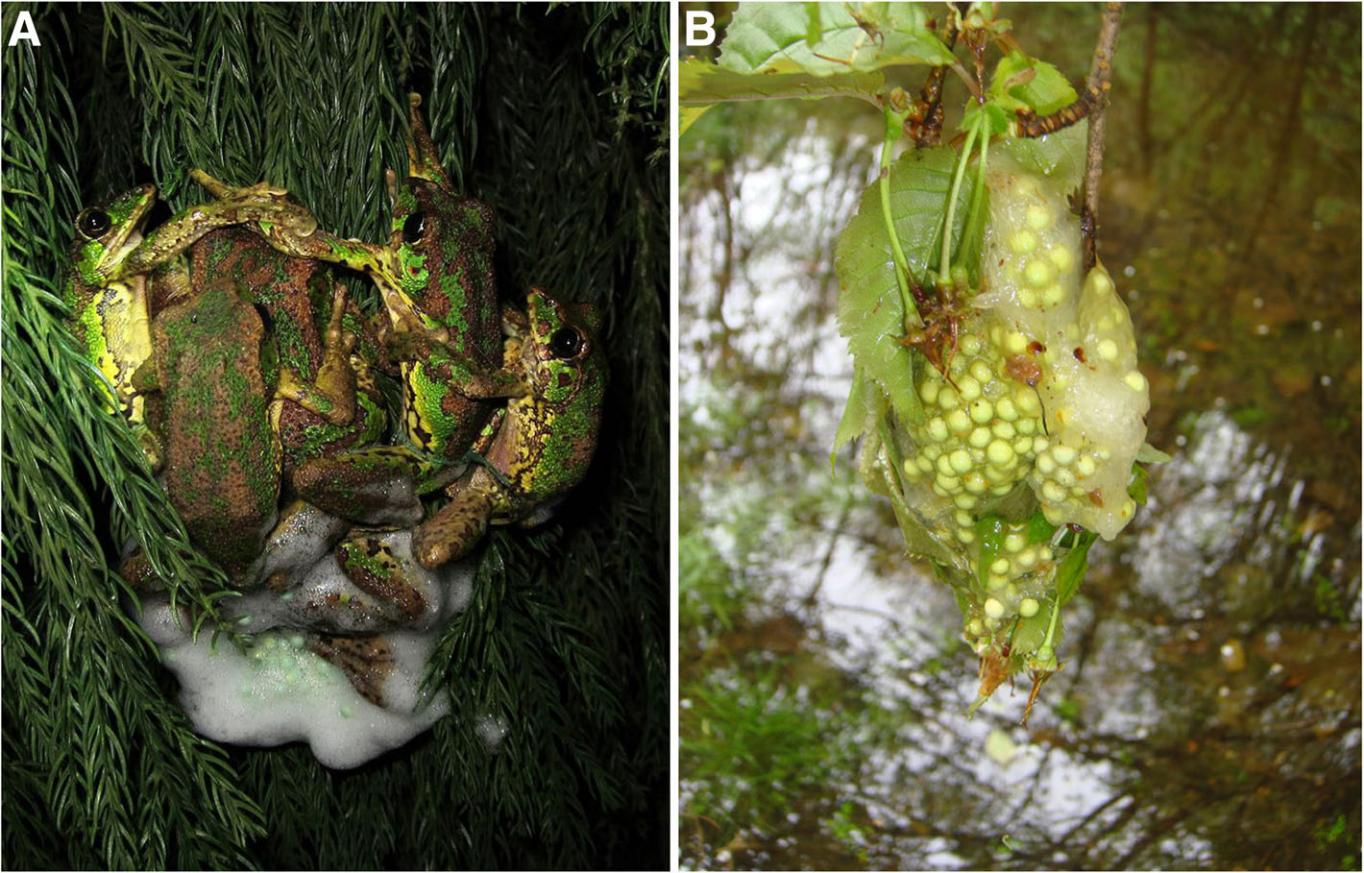
The leaf foam nest of *Rhacophorus omeimontis*. **a**. A spawning group of *R. omeimontis* were producing the foam nest. **b**. A finished foam nest on the leaves

The Omei treefrog, *Rhacophorus omeimontis* (Anura: Rhacophoridae), presents a foam-nesting strategy during breeding seasons, and females commonly mate with multiple males in one spawning group [21–23]. During each breeding season, males first arrived at a permanent pond surrounded by vegetation. The female is usually grasped by a male at the time of arrival and then moves to find an appropriate spawning site, usually on a leaf above the water surface [22, 23]. The female releases a clutch of eggs together with foam precursor fluid; at the same time, the paired male expels the sperm and, using a rapid “egg-beater” motion of the legs, whips the mixture into a white foamy mass containing the fertilized eggs. Then, other males gradually join the mating pair, forming a spawning group (Fig. 1A). At the end of spawning, the males leave the mating group, and the female remains until leaf nesting is finished [23–25]. After the mating period, the foam nests remain on the vegetation or leaves for almost one month (Fig. 1B). The eggs and embryos in the foam nests commonly exhibit a very low mortality [21, 23]. Therefore, *R. omeimontis* may be an appropriate model for studying the molecular basis of arboreal foam nest production in anuran species.

Transcriptome sequencing is an efficient way to examine the global molecular response of living organisms to the conditions of certain life history periods [26, 27]. Differential gene expression analysis provides a method for studying the molecular basis of context-specific tissue activity without the need for a reference genome [27, 28]. In this study, we performed transcriptome sequencing of the oviduct of female *R. omeimontis* from the stage when foam nest production was finished (AFNP_O) to the stage before foam nest production (BENP_O). Then, we compared the differential gene expression profiles of the oviducts between these two periods of foam nest production. Our aim was to investigate genes and pathways that may play important roles during the production of leaf foam nests.

## Results

### De novo transcriptome sequencing of *R. omeimontis*

To investigate the expression changes of genes in the oviduct of female *R. omeimontis* during the production of foam nests, we performed transcriptome sequencing of *R. omeimontis* oviducts during two periods (AFNP_O and BENP_O) with three biological replicates in each period. Due to the absence of a reference genome for *R. omeimontis*, we de novo assembled a transcriptome as a reference for read mapping and gene expression profiling in this species. Illumina sequencing of the *R. omeimontis* oviducts generated a total of 188,814,226 raw reads. After removing adapters, primers and low-quality reads, we obtained a total of 182,618,614 clean reads for the six samples. The sequencing data had an average Q20 of 96.71% and GC content of 46.43% (Additional file 1: Table S1). The clean reads were then assembled into transcripts using Trinity [29]. In the assembly of *R. omeimontis*, 146,672 transcripts with a total length of 162,215,340 bp were obtained (Table 1). These transcripts were finally assembled into 84,917 unigenes with a total length of 69,985,136 bp. The N50 lengths of the transcripts and unigenes were 2185 bp and 1631 bp, respectively. Most of the unigenes (51,358, 60.48%) were 200–500 bp in length (Additional file 2: Figure S1). A detailed summary of the transcriptome data can be found in Table 1. To verify the assembly quality, we mapped the clean reads to the assembled unigenes, and the mapping rates for the six samples ranged from 77.62 to 81.92% (Additional file 1: Table S1). Taken together, these metrics indicate the high quality of our transcriptome data, which is the foundation for all subsequent analyses [30]. Importantly, this study represents the first transcriptome exploration of oviducts in *R. omeimontis* and provides valuable gene expression information that can be used to further understand the genetic basis of foam nest production in this species.

**Table 1:** Summary information for transcriptome assembly in *Rhacophorus omeimontis*

Length	Transcripts	Unigenes
200–500 bp	70,495	51,358
500–1000 bp	28,707	14,839
1000–2000 bp	23,711	10,099
> 2000 bp	23,759	8621
Total number	146,672	84,917
Total length	162,215,340	69,985,136
Mean Length	1106	824
N50	2185	1631

### Functional annotation and classification of the unigenes

To explore the potential function of the unigenes in the *R. omeimontis* oviduct during foam nest production, we annotated the unigenes against the NR, Swiss-Prot, Pfam, GO, Clusters of EuKaryotic (KOG) and Kyoto Encyclopedia of Genes and Genomes Orthology (KO) databases. Of the 84,917 unigenes, 28,681 unigenes (33.77%) presented a positive match against at least one database (Table 2). Thus, many of the unigenes have not been annotated, which may be partly due to the limited genetic resources for non-model amphibian species, especially genetic data on expression in the oviduct tissue. Furthermore, unigenes with partial or misassembled transcripts, sequences from untranslated regions (UTRs) of proteins and meaningless proteins could not have a match to any genes. These reasons may explain the annotation failure for those unigenes. The summary annotation information of *R. omeimontis* unigenes was provided in Table 2.

**Table 2:** Summary of the annotation information among unigenes in *Rhacophorus omeimontis*

Database	Annotated Unigenes	Percentage (%)
NR	22,590	26.60
Swiss-Prot	19,780	23.29
Pfam	20,685	24.35
GO	22,133	26.06
COG	11,413	13.44
KEGG	10,735	12.64
All	28,681	33.77

NR, NCBI non-redundant protein sequences database; Swiss-Prot, A manually annotated and reviewed protein sequence database; Pfam, Protein family database; GO, Gene Ontology database; KEGG, Kyoto Encyclopedia of Genes and Genomes database; COG, Clusters of Orthologous Groups of proteins database; All, Total number of unigenes that successfully annotated in at least one database

**Fig. 2:**
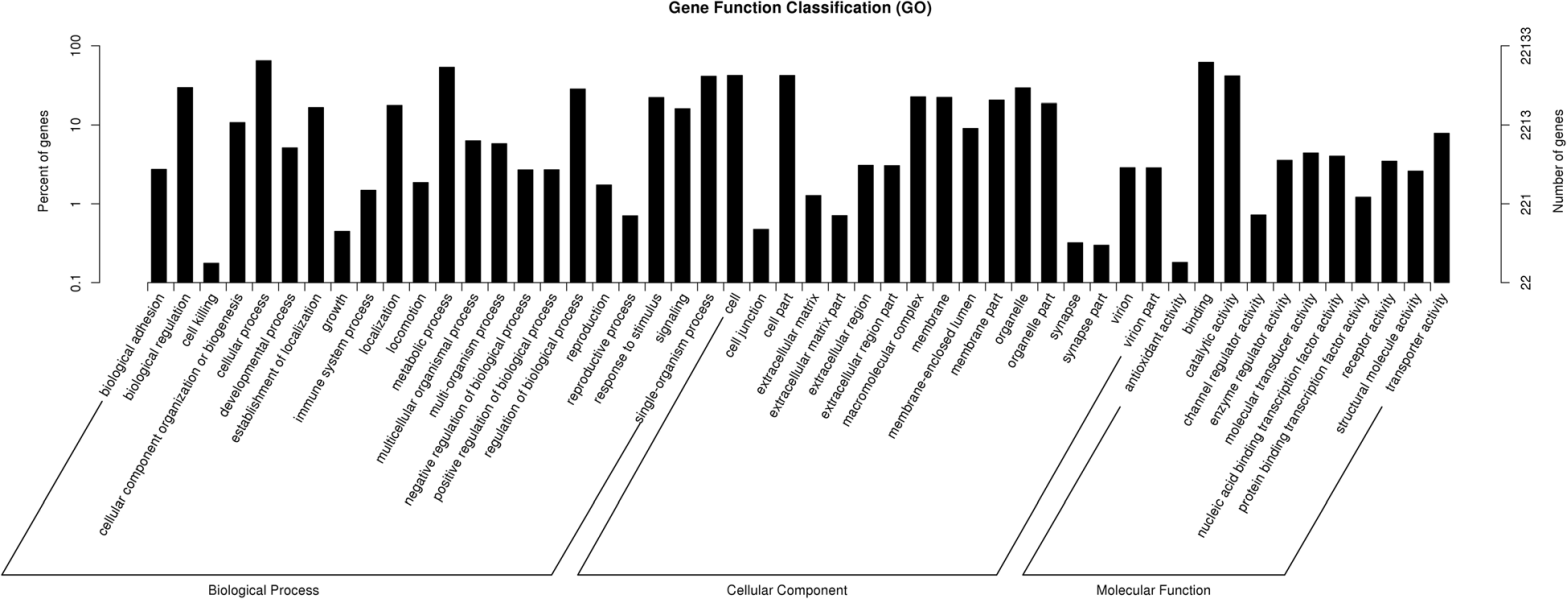
Gene Ontology classification of the *Rhacophorus omeimontis* unigenes

In GO classification, a total of 22,133 unigenes (26.06%) were assigned to 55 sub-categories of GO terms belonging to the three main categories: Biological Process, Cellular Component and Molecular Function (Fig. 2). The category Biological Process contained 22 sub-categories, and cellular process (64.66%) and metabolic process (53.40%) were the two main enriched GO terms in this category (Fig. 2). The Cellular Component category included 17 sub-categories, and the two most enriched GO terms were cell (42.11%) and cell part (42.05%). In category Molecular Function, 11 sub-categories were found, and the most enriched term was binding (61.78%), followed by the term of catalytic activity (41.51%; Fig. 2).

**Fig. 3:**
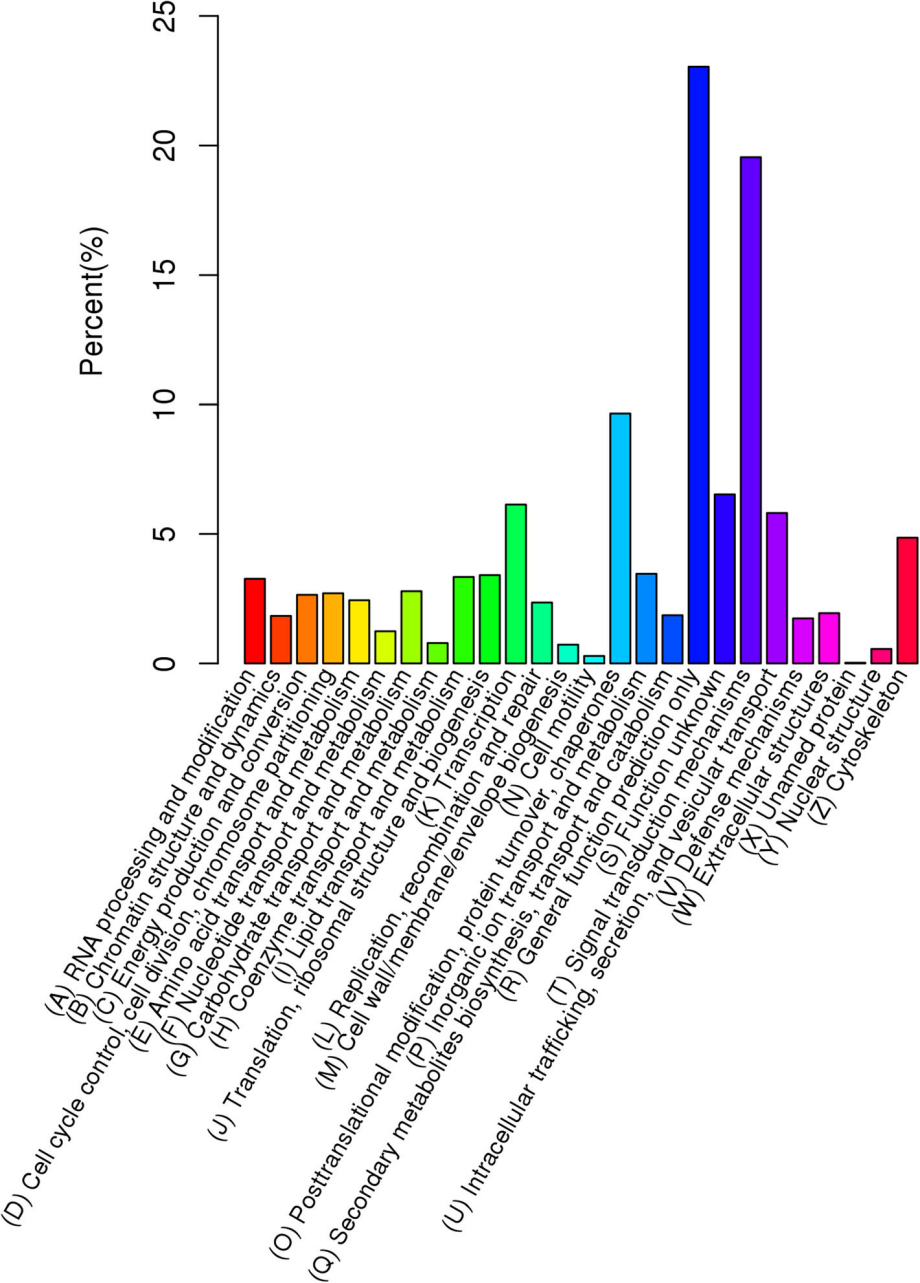
Clusters of EuKaryotic Orthologous Groups (KOG) classification

The COG database mainly contains the phylogenetic classification of proteins encoded in 21 complete genomes of bacteria, archaea and eukaryotes [31]. In the eukaryote-specific COG (KOG) classification, a total of 11,413 unigenes (13.44%) were functionally classified into 25 KOG categories (Fig. 3). Among all KOG classifications, General function prediction only (R) occupied the largest proportion (23.04%), followed by the Signal transduction mechanisms (S; 19.55%) and Posttranslational modification, protein turnover, chaperones (O; 9.66%).

**Fig. 4:**
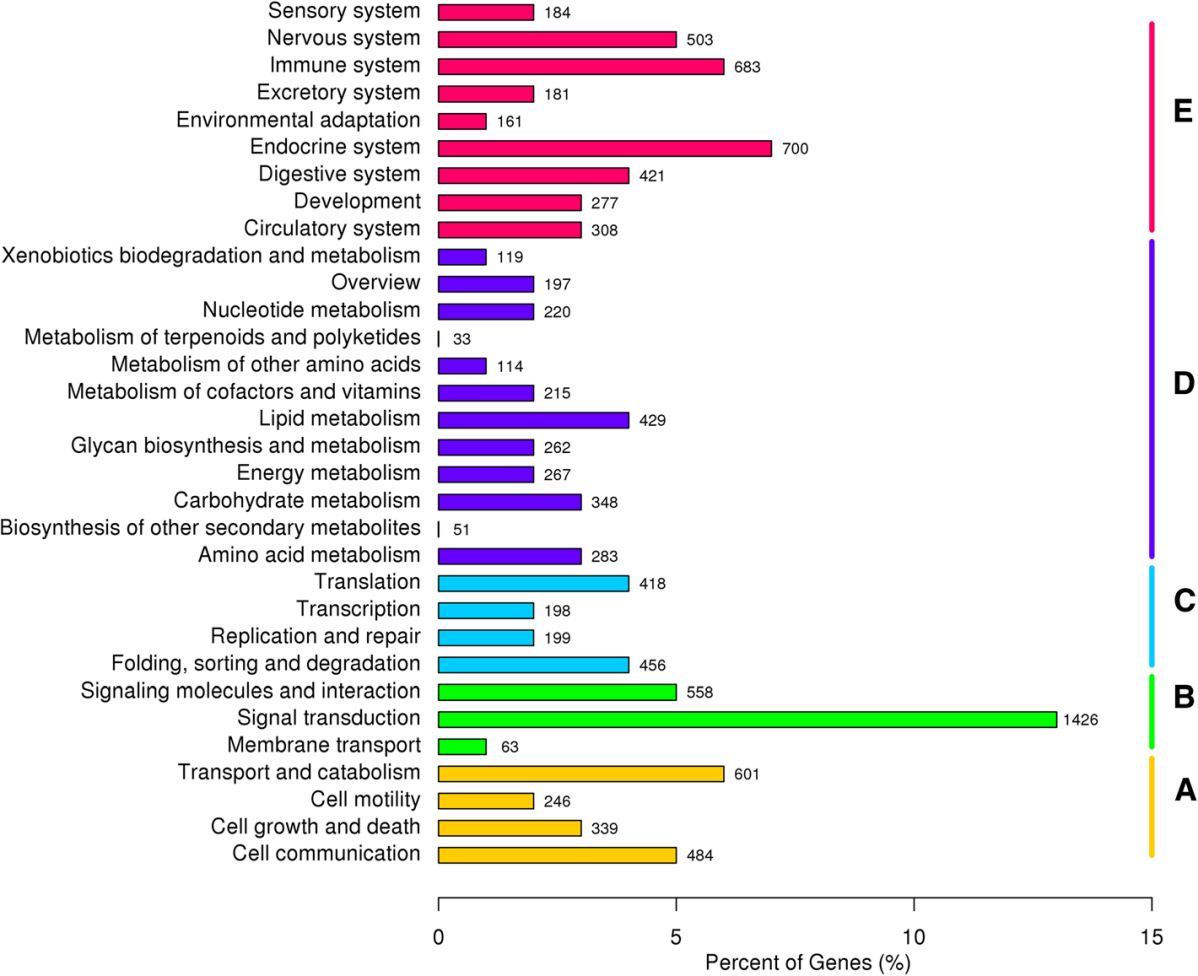
KEGG classification of the unigenes in *Rhacophorus omeimontis*. A to E indicate five categories of KEGG pathways: **a**, Cellular processes; **b**, Environmental information processing; **c**, Genetic information processing; **d**, Metabolism; **e**, Organismal systems

To further understand the gene functions involved in the metabolic pathways, we annotated all unigenes against the KEGG database. A total of 10,735 unigenes (12.64%) were mapped into 263 signaling pathways belonging to 32 categories of pathway hierarchy level 2 and five categories of hierarchy level 1 (Fig. 4). Among the 32 pathways, Signal transduction (13.28%), Endocrine system (6.52%) and Immune system (6.36%) were the most enriched pathways. These annotations will provide valuable resources for further studies of *R. omeimontis*.

### Analysis of differentially expressed genes

**Fig. 5:**
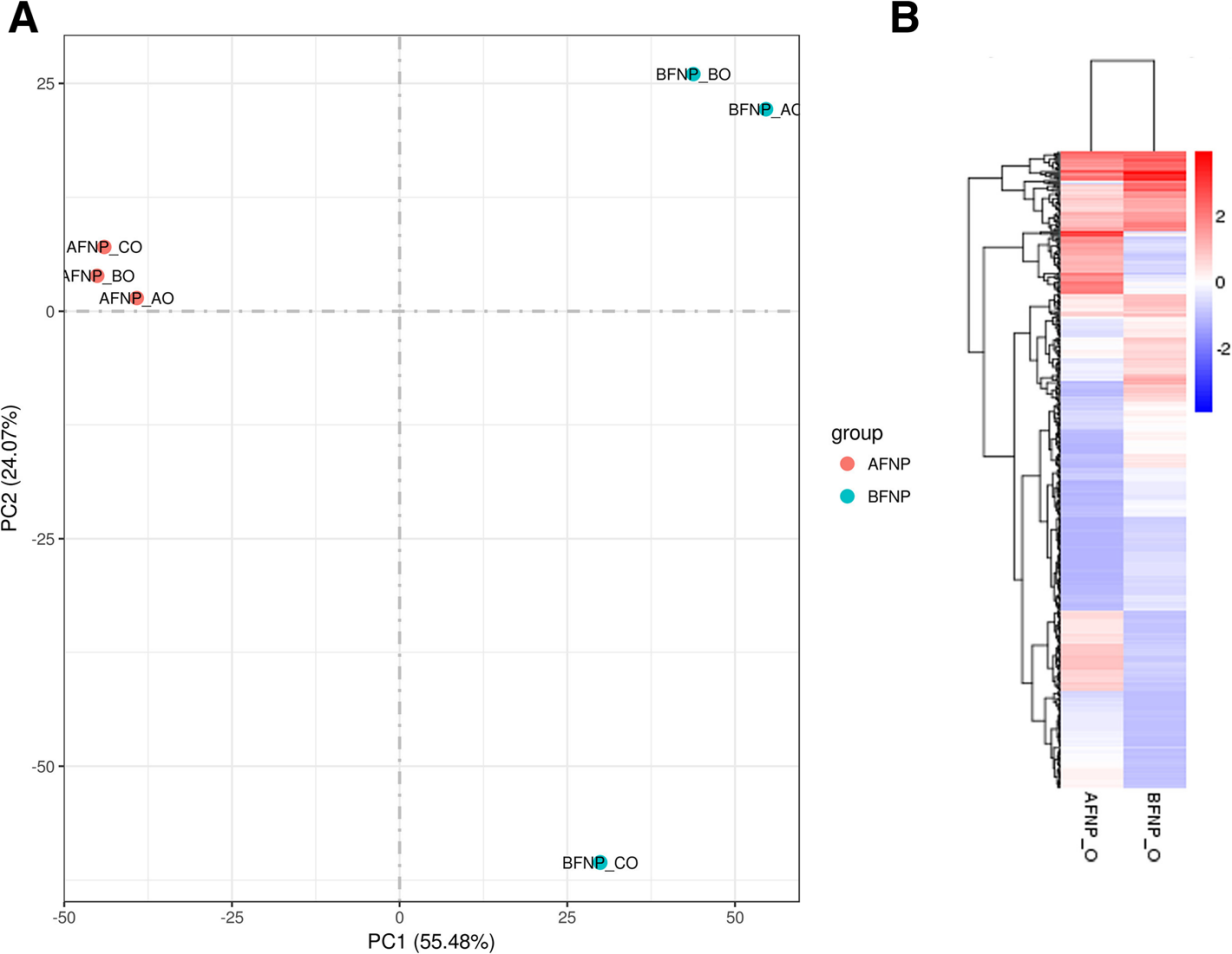
Analysis of transcriptome data for six oviduct samples from the periods before and after foam nest construction in *Rhacophorus omeimontis*. **a**. Principal component analysis (PCA) of six oviduct transcriptome data sampled from two periods of foam nest construction. The first principal component (PC1) accounts for 55.48% of the variation, while the second principal component (PC2) accounts for 24.07% of the variation. **b**. Hierarchical clustering analysis of differentially expressed genes in oviduct between the two periods during foam nest construction. The intensity of colors indicates the expression levels of the genes

**Fig. 6:**
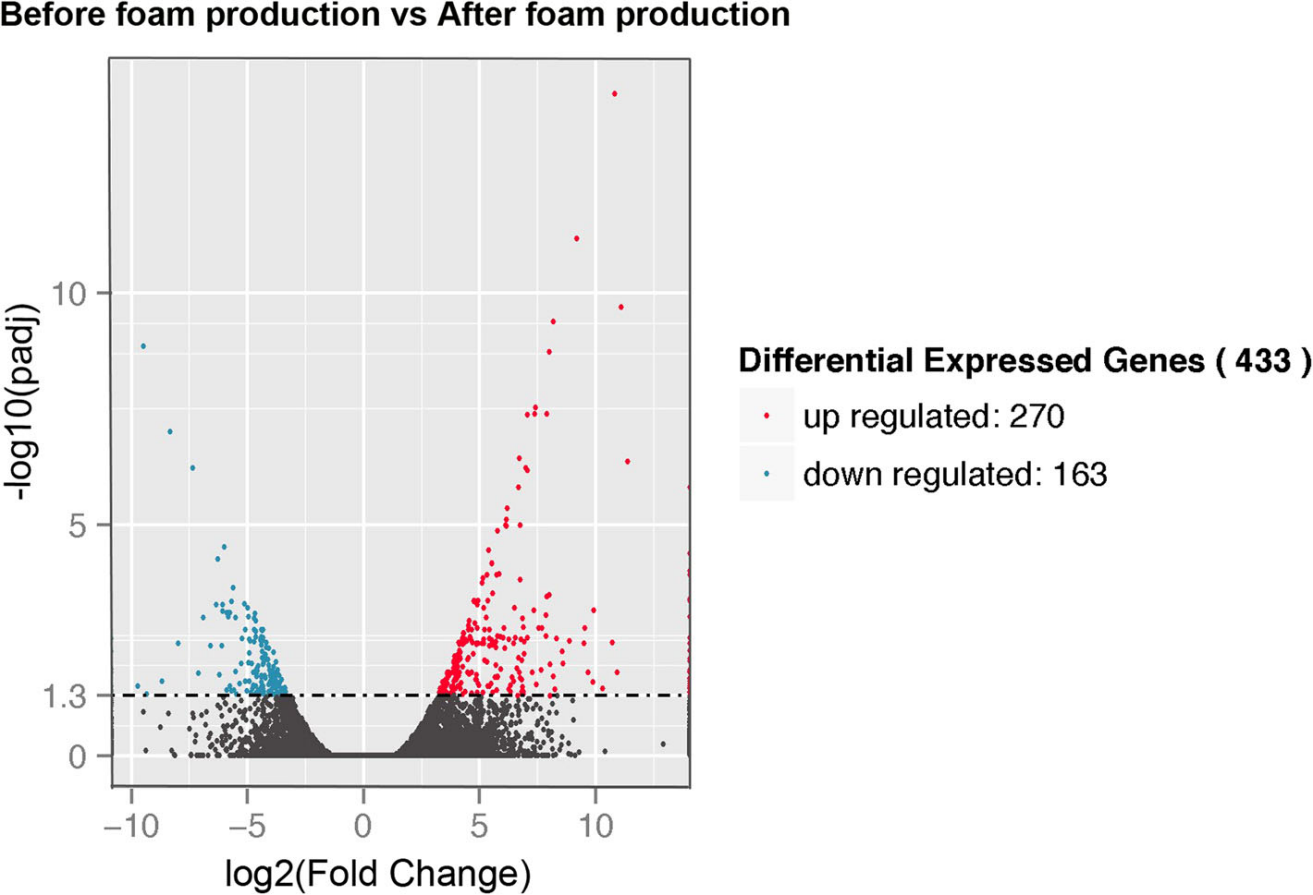
The differentially expressed genes in the oviduct between the two periods during foam nest construction in *Rhacophorus omeimontis*

**Fig. 7:**
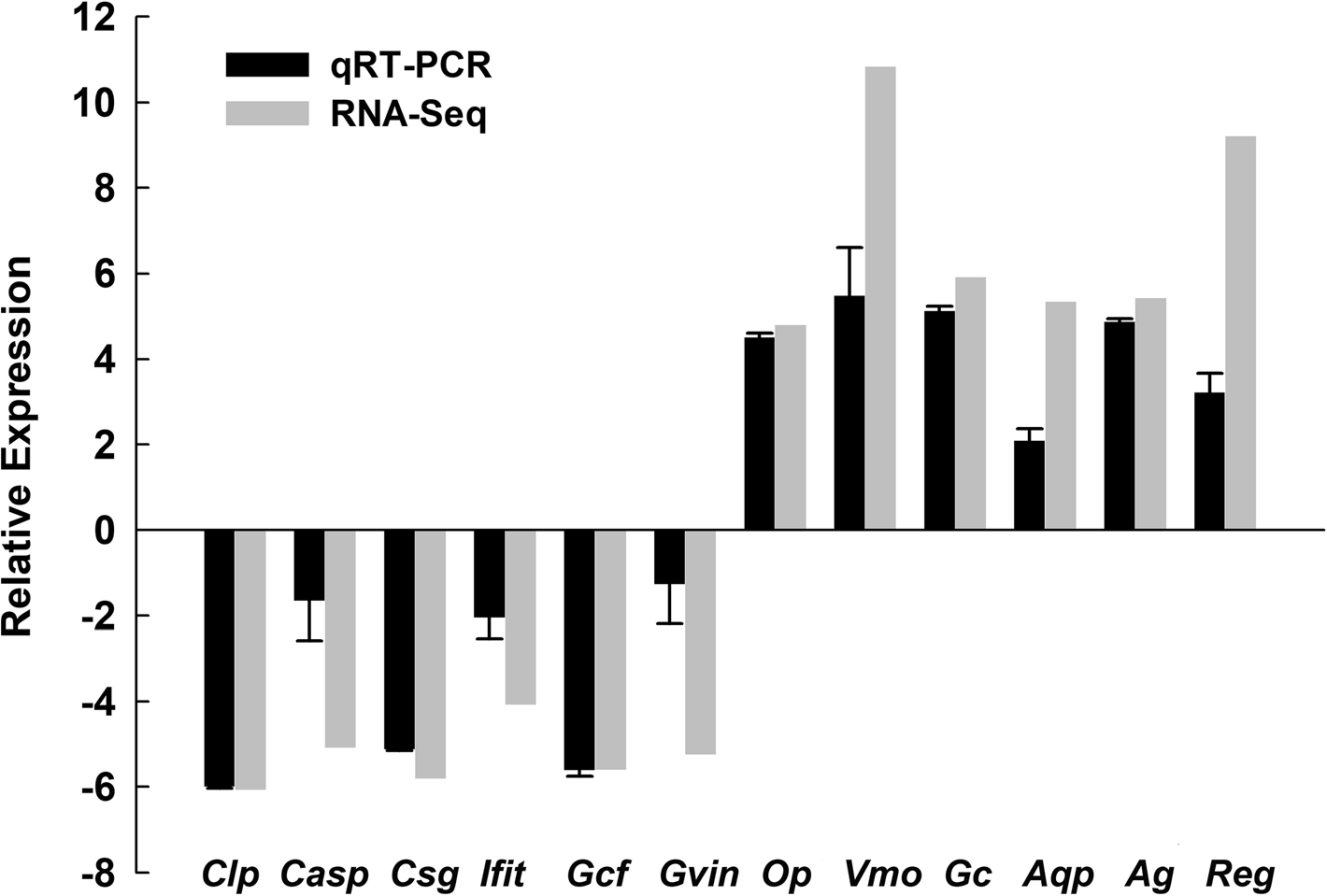
Quantitative real-time PCR confirmation of the differentially expressed genes identified by RNA-seq

To explore DEGs between the two periods of foam nest construction, we estimated the gene expression levels of each sample by RSEM and calculated the FPKM value. The whole transcriptomes of oviduct between the different periods were first analyzed by a principal component analysis. The first principal component (PC1, accounting for 55.48% of the variation) and the second principal component (PC2, accounting for 24.07% of the variation) together explained 79.55% of the variance in the transcriptome data (Fig. 5A). Principal component analysis revealed strong clustering between the two periods of foam nest construction. In addition, the global gene expression profiles of oviduct from the two periods also showed different patterns. The final set of DEGs in oviduct between the two periods was revealed by the hierarchical clustering of expression patterns (Fig. 5B). In total, we identified 433 DEGs in the oviduct of *R. omeimontis*, among which 270 genes were upregulated and 163 genes were downregulated during the AFNP_O period compared with the BFNP_O period (Fig. 6; Additional file 1: Table S2, Table S3). Among all DEGs, 259 (59.82%) were successfully annotated. To confirm the differential expression profiles obtained with the transcriptome data, we selected a total of 14 genes from DEGs for qRT-PCR analysis. The result revealed that 12 out of 14 genes closely matched the results of RNA-seq, with a correlation coefficient *R* of 0.92 (Fig. 7).

**Fig. 8:**
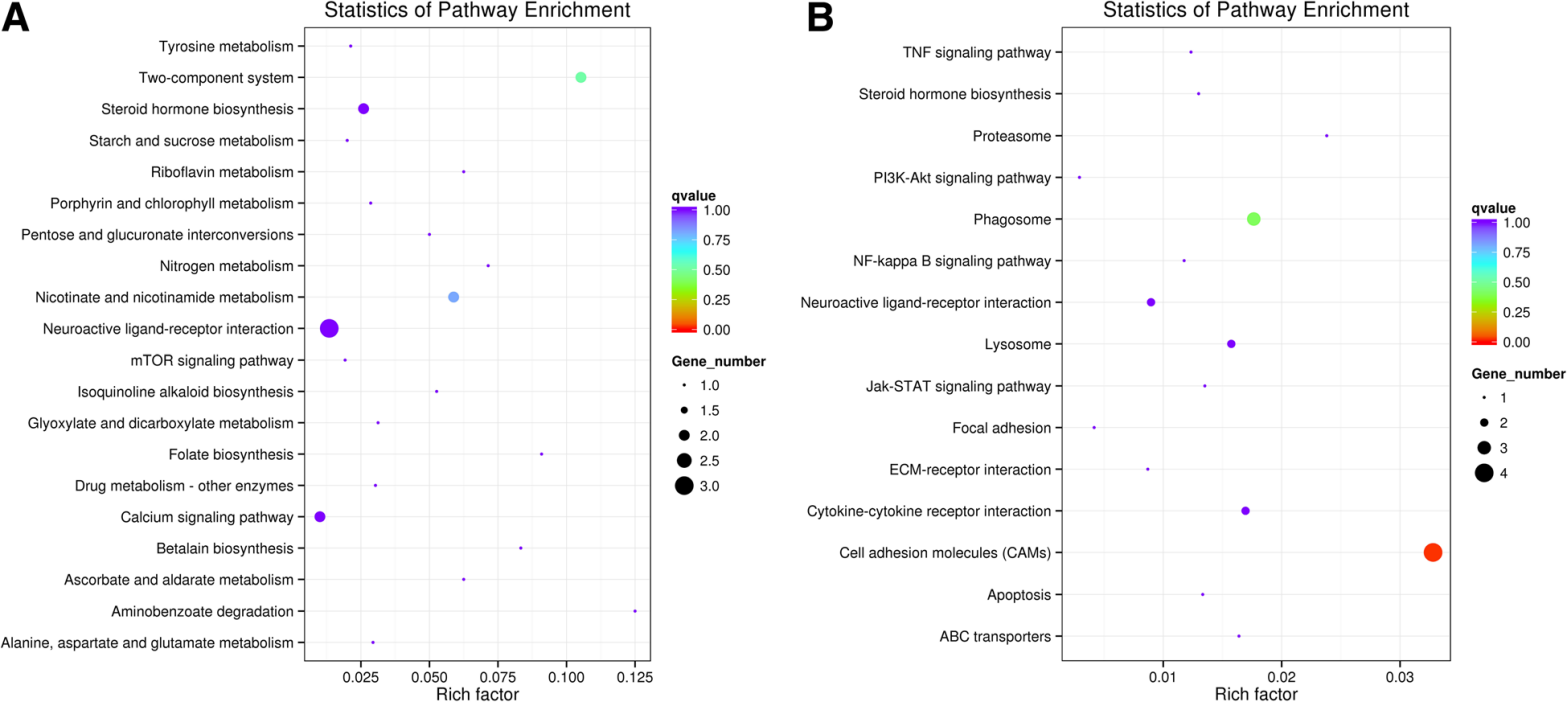
KEGG significant enrichment analysis of differentially expressed genes (DEGs) between the two periods of foam nest construction. **a**. KEGG significant enrichment analysis of the upregulated DEGs. **b**. KEGG significant enrichment analysis of the downregulated DEGs

To better understand the potential biological functions and metabolic pathways that the DEGs were involved in during the two periods of foam nest production, we compared all DEGs to the GO and KEGG databases and applied the significant enrichment analysis. In the upregulated DEGs, extracellular space (GO:0005615) and extracellular region (GO:0005576) were the significantly enriched GO terms (Additional file 1: Table S5; Additional file 2: Figure S2). However, no significant enrichment of GO terms was identified in the downregulated DEGs (Additional file 1: Table S6). Furthermore, all 433 DEGs were classified into 46 KEGG pathways. Among these pathways, the ‘two-component system’, ‘cell adhesion molecules’, ‘steroid hormone biosynthesis’, ‘neuroactive ligand-receptor interaction’, ‘nicotinate and nicotinamide metabolism’ and ‘cytokine-cytokine receptor interaction’ pathways were significantly enriched (Fig. 8). Generally, these significantly enriched pathways were mostly basic metabolism-related pathways. In contrast, in the upregulated DEGs, the significantly enriched KEGG pathways were ‘two-component system’, ‘nicotinate and nicotinamide metabolism’, ‘aminobenzoate degradation’, ‘steroid hormone biosynthesis’ and ‘folate biosynthesis’ (Additional file 1: Table S7; Fig. 8). These DEGs were mostly related to biological processes of reproduction and sex hormone biosynthesis. In the downregulated DEGs, the significantly enriched KEGG pathways were ‘cell adhesion molecules’, ‘phagosome’, ‘cytokine-cytokine receptor interaction’ and ‘lysosome’ (Additional file 1: Table S8; Fig. 8). Therefore, the downregulated DEGs were predominantly participate in pathways with immunity-associated functions.

We further identified the top ten upregulated and top ten downregulated annotated DEGs in the oviduct based on the order of FDRs to obtain potential genes that may directly control the construction or composition of the foam nest (Table 3, Additional file 1: Table S2, Table S3). Among these top 20 annotated DEGs, ten genes were immune-related genes, including REG4, CPAMD9, LYZ2, IL17B, PARP14, CD116, MHC I and C7 (Table 3). Of the top ten upregulated DEGs, three were Regenerating islet-derived protein 4-like genes (REG4, Table 3). And VMO1 gene was the most differentially expressed (log2FC = 10.83, FDR = 4.99E-15, Table 3) of the upregulated genes. Among the top ten downregulated genes, two genes were related to lipid metabolism, including APOC-1 and CYP11A1 gene. And four DEGs were related to immune responses, including MHC I, PARP14, C7 and CD116 gene. In addition, mmp18 gene were also among the top ten downregulated genes.

**Table 3:** Top ten upregulated and top ten downregulated genes in the oviduct between the period before (BFNP_O) and after (AFNP_O) the foam nest construction in *Rhacophorus omeimontis*

Gene ID	BFNP_O FPKM	AFNP_O FPKM	log2 FC	FDR	Gene description
Upregulated genes					
comp102470_c0	83,413.99956	45.86109708	10.829	4.99E-15	Vitelline membrane outer layer protein 1 homolog precursor (VMO1)
comp105433_c0	1,464,303.449	2473.865279	9.2092	6.67E-12	Regenerating islet-derived protein 4-like (REG4)
comp116497_c3	282,853.1745	960.5393083	8.202	4.19E-10	C3 and PZP-like alpha −2- macroglobulin domain-containing protein 9 (CPAMD9)
comp86479_c0	240,749.3995	927.3587874	8.0202	1.83E-09	Lysozyme C-2 precursor (LYZ2)
comp86445_c0	52,409.4277	19.36436651	11.402	4.27E-07	Hypothetical protein
comp63781_c0	370.8359078	2.766443957	7.0666	6.62E-07	Interleukin 17B precursor (IL17B)
comp93501_c0	8951.186584	86.50103654	6.6932	1.57E-06	Regenerating islet-derived protein 4-like (REG4)
comp112645_c0	975.690657	13.13121608	6.2154	4.51E-06	LOC100158309 protein
comp111158_c1	410.1014732	3.763068643	6.7679	1.06E-05	ORF2
comp93501_c1	46,423.55193	845.0879313	5.7796	1.39E-05	Regenerating islet-derived protein 4-like (REG4)
Downregulated genes					
comp83066_c0	17.63586872	12,383.38172	−9.4557	1.37E-09	Apolipoprotein C-I-like (APOC-1)
comp86958_c0	15.29489023	975.5912786	−5.9952	3.04E-05	Matrix metalloproteinase-18 (mmp18)
comp93961_c0	2.611183539	200.4585611	−6.2625	5.69E-05	Receptor-transporting protein 3 (Rtp3)
comp110795_c0	5.596429558	271.7805192	−5.6018	0.000229	Granulocyte-macrophage colony-stimulating factor receptor subunit alpha (CD116)
comp115970_c0	81.27326898	5454.637376	−6.0686	0.000528	Poly [ADP-ribose] polymerase 14-like (PARP14)
comp98850_c0	12.0415714	978.7260655	−6.3448	0.000528	MHC class I antigen, partial (MHC I)
comp112386_c0	13.56931178	432.0370548	−4.9927	0.000625	Hypothetical protein
comp105453_c0	1.470656838	98.15940537	−6.0606	0.000742	Cytochrome P450 family 11 subfamily A polypeptide 1 (CYP11A1)
comp108188_c0	2.582748907	137.2794339	−5.7321	0.00079	RING finger protein 213-like (RNF213)
comp107251_c1	35.30863025	898.6047342	−4.6696	0.000826	Complement component 7 precursor (C7)

## Discussions

The production of foam nests is one of the strategies that has evolved to allow some anuran species to protect their eggs and larvae. Despite considerable knowledge of the biochemical components of anuran foam nests, little is known about the molecular basis involved in the construction of foam nest [5–7]. In this study, we performed a transcriptome of oviduct in female *R. omeimontis* from two different periods during foam nest production to explore genes and pathways that may play important roles in this process.

Differential expression analysis revealed 433 DEGs between the two periods during foam nest production. Of these genes, 270 were upregulated and the remaining 163 genes were downregulated. The results from GO and KEGG analysis indicated that various numbers of DEGs were involved in different processes of extracellular metabolism and steroid hormone biosynthesis. The upregulated DEGs were mostly related to biological processes of sex hormone biosynthesis. Before foam nest production began, the females were in the active breeding period; therefore, the significantly enriched pathways in the oviduct tissue were associated with reproduction and sex hormones, such as ‘folate biosynthesis’ and ‘steroid hormone biosynthesis’. Folate, a kind of vitamin B, is an indispensable element of DNA methylation that plays a key role during the process of embryogenesis. A shortage of folic acid may induce neural tube defects [32, 33].

The downregulated DEGs were predominantly participate in pathways with immunity-associated functions. Phagosomes, lysosomes and proteasomes play a part in antigen processing and presentation [34–37]. Apoptosis is important for the development and normal functioning of the immune system [38]. The TNF signaling pathway, Jak-STAT signaling pathway, NF-kappa B signaling pathway, and PI3K-Akt signaling pathway are activated during the process of immune response, and the cytokine-cytokine receptor interaction contributes to the mediator roles of these signaling pathways [39–45].

Among the top 20 annotated DEGs, nine genes were immune-related genes, indicating that immune function plays a key role in the production of the leaf foam nest. Of the top ten upregulated DEGs, three were REG4 gene. REG4 is a member of the regenerating (Reg) gene family, which belongs to the calcium-dependent lectin gene superfamily [46]. Lectins are a kind of carbohydrate-binding family proteins that are widely found in viruses, bacteria, plants, animals and humans [47, 48]. Studies on the túngara frog (*Engystomops pustulosus*) indicated that the foam nest was a mixture of six proteins, named ranaspumins (Rsn-1 to Rsn-6); four of these proteins exhibit lectin activity and may play important roles in forming long-term foam stabilization by contributing to the formation of a cross-linked carbohydrate network at the air-water interface and defending against pathogen attacks [17]. Rsn-3 to Rsn-5 have amino acid sequences similar to those of fucolectins that predominately bind to fucose, whereas Rsn-6 belongs to the C-type lectins that are always associated with galactose binding [17]. Lectins bind to molecular patterns of pathogens, and they hinder microbial dissemination by agglutinating granules with a specific array of carbohydrates [49]. In addition, lectins participate in the process of embryonic development and intracellular glycoprotein transport in some pathways [50–52]. Therefore, the lectins may be the constituent proteins of the leaf foam nest of *R. omeimontis*, and this component was also found in the water-based foam nest of the túngara frog [17].

The VMO1 gene was the most differentially expressed of the upregulated genes. A previous study revealed that VMO1 reduced the surface tension of tears and produced a more stable tear film in vivo [53]. Thus, we considered that VMO1 in the oviduct of *R. omeimontis* may have a similar function that could reduce the surface tension of foams and produce a more stable and biocompatible foam nest environment. However, compared to other anuran species with a water-interface foam nest, *R. omeimontis* did not exhibit similar genes encoding protein surfactants that could reduce surface tension and enable foam nest formation, such as Rsn-1, Rsn-2, ranasmurfin gene and Lv-Rsn-1 [17, 54–57]. Considering that the composition of the foam nest should be species-specific, the arboreal foam nest constructed by *R. omeimontis* may have different types of surfactant proteins that differ from those in aquatic foam nests. Moreover, published genetic data for anuran species on the expression in the oviduct are still limited; therefore, many of our unigenes have not been functionally annotated, and these genes may include surfactant protein genes or other important genes associated with foam nest formation in *R. omeimontis*.

Of the top ten downregulated genes, two genes were related to lipid metabolism, including APOC-1 and CYP11A1 gene. APOC-1 encodes a component of lipoproteins that plays a central role in high density lipoprotein (HDL) and very low-density lipoprotein (VLDL) metabolism of vertebrates [58, 59]. Previous studies suggest that overexpression of APOC-1 in humans and transgenic mice may cause high levels of triglycerides (TGs) as a consequence of APOC-1 inhibition activity of lipoprotein lipase (LPL)-mediated TG lipolysis [60, 61]. LPL hydrolyses the TGs into free fatty acids (FFAs), which are used for energy metabolism or storage in tissues [62–64]. The reproductive process in anurans is an energy-consuming activity, especially during the amplexus and spawning period [65, 66]. Additionally, the foams are energetically expensive to make and difficult to maintain, with a tendency to collapse over time unless stabilized mechanically or kinetically by additional processes [56, 67]. Based on our field observation, female *R. omeimontis* built their foam nest by releasing cloacal mucus fluid and whipping the blend into foam by churning her back legs, and this process commonly takes two to three hours. Thus, we hypothesized that foam nest building is an energy-consuming process for female *R. omeimontis*, and the highly expressed lipid metabolism-related genes during AFNP_O period may be involved in this process.

CYP11A1 encodes a member of the cytochrome P450 superfamily catalyzing the conversion of cholesterol to pregnenolone [68]. This is the first reaction in the process of steroidogenesis in all mammalian tissues that specialize in the production and metabolism of various steroid hormones [68, 69]. Matrix metalloproteinases (MMPs) play an important role in extracellular matrix remodeling and degradation during organ development and pathological processes [70]. In *Xenopus laevis*, mmp18 may play a role in larval tissue degeneration and adult organogenesis during metamorphosis [71, 72]. The highly expressed CYP11A1 and mmp18 during AFNP_O period in the oviduct of female *R. omeimontis* may be important for regulating foam-nesting activity through the steroid hormone pathway and extracellular matrix proteolysis.

Additionally, four immune-related genes were also found in the top ten downregulated genes. Major histocompatibility complex (MHC) molecules vitally participate in immune response by presenting antigens to T cells [73, 74]. The PARP14 gene encodes a protein of poly (ADP-ribose) polymerase family member 14. Poly (ADP-ribose) polymerase is an immediate DNA damage-dependent post-translational modifier of histones and other nuclear proteins that contributes to the survival of injured proliferating cells [75, 76]. The C7 gene encodes a serum glycoprotein that forms a membrane attack complex together with the complement components C5b, C6, C8, and C9 as part of the terminal complement pathway of the innate immune system [77]. CD116 is a receptor for granulocyte-macrophage colony-stimulating factor, which stimulates the production of white blood cells [78]. In anuran amphibians, embryos and larvae are vulnerable to bacterial and fungal pathogen infections [79–81]. To protect the offspring, females would maternally transfer antibodies to offspring for immune defense before the larvae are immune at maturity [82, 83]. Therefore, the highly expressed immune-related genes in the oviduct of *R. omeimontis* could be important in the production of foam nests for protecting the eggs and embryos.

Overall, the above-mentioned candidate genes from the oviduct of *R. omeimontis* encoded and regulated a range of proteins with a mixture of surfactant, carbohydrate-binding and immune defense activities that together provide a stable, biocompatible, protective foam environment for developing eggs and embryos. The steroid hormone pathway and lipid metabolism may play important roles in regulating the process of foam nest construction.

## Conclusions

In this study, transcriptome analysis was performed with the oviduct tissue of *R. omeimontis* to explore the molecular basis of leaf foam nest construction. Our transcriptome data provided a rich list of genes expressed during the two different periods of foam nesting. A total of 433 DEGs were identified, with 270 genes being upregulated and 163 genes being downregulated. In summary, numerous functional genes and metabolic pathways were likely associated with the process of foam nest construction. During the period when foam nest production began, genes encoding lectin, surfactant protein and immune components were highly expressed, as their products were indispensable components of the foam nest. During the period when the foam nest production was finished, genes related to lipid metabolism, steroid hormone and immune function were highly expressed, and these genes may be important for regulating the process of foam nesting. Our findings will provide useful information for further investigation of anuran foam nest construction at the molecular level.

## Methods

### Sample collection and RNA extraction

The female *R. omeimontis* individuals were obtained from the population in Badagong Mountain Natural Reserve in Hunan Province, central China (29°47′02″ N, 110°05′27″ E), during the breeding season. The sampling was performed during the period when foam nest production began (BENP_O) and the period after foam nest production was finished (AFNP_O). During each sampling period, three females were caught from groups in amplexus and designated as biological replicates. In BENP_O period, we sampled the oviducts of females immediately when the female that was gripped by a male arrived at the breeding and spawning tree. This period could represent the preparation or beginning stage of foam nest production. In the AFNP_O period, we sampled the oviducts of females immediately when the male left the amplexus. Commonly, females would stay with the foam nest for about half an hour, and continue whipping the foam and wrapping the foam nest with surrounding leaves. Thus, this period could represent the active phase of foam nest construction. In both sampling procedures, we caught females as soon as possible and kept them in individual plastic boxes containing leaves from their natural habitats. The living frogs were euthanized by injecting buffered MS-222 solution and dissected immediately with 0.1% diethyl pyrocarbonate (DEPC)-ddH_2_O solution-treated scissors. The tissue samples were taken from the oviduct of the frogs and stored in liquid nitrogen until needed.

Total RNA from each oviduct sample was extracted using an RNA extraction kit (Omega Bio-Tek, USA) according to the manufacturer’s instructions. The quality of the total RNA was assessed by 1% gel electrophoresis, and the RNA purity was checked using a NanoPhotometer® spectrophotometer (IMPLEN, CA, USA). We measured the RNA concentration using a Qubit® RNA Assay Kit in a Qubit® 2.0 fluorometer (Life Technologies, CA, USA). RNA integrity was checked using a RNA Nano 6000 Assay Kit of the Agilent Bioanalyzer 2100 system (Agilent Technologies, CA, USA). Total RNA was extracted separately from each oviduct sample for three replicates in each studied period, and six high-quality RNA samples were obtained.

### cDNA library construction and Illumina sequencing

A total of 3 μg of RNA pre-sample was used as input material for cDNA library construction. cDNA libraries were generated using an NEBNext® Ultra™ RNA Library Prep Kit for Illumina® (NEB, USA) following the manufacturer’s recommendations. In brief, mRNA was purified from total RNA using poly-T oligo-attached magnetic beads. Fragments were generated using divalent cations under elevated temperature in NEBNext First Strand Synthesis Reaction Buffer (5X). First-strand cDNA was synthesized using a random hexamer primer and M-MuLV Reverse Transcriptase (RNase H^−^). Second-strand cDNA synthesis was subsequently performed using DNA polymerase I and RNase H. To select cDNA fragments of preferentially 150~200 bp in length, the library fragments were purified with the AMPure XP system (Beckman Coulter, Beverly, USA). PCR was then performed with Phusion High-Fidelity DNA polymerase, universal PCR primers and Index (X) Primer. PCR products were purified using the AMPure XP system, and library quality was assessed with the Agilent Bioanalyzer 2100 system.

The cDNA samples were then clustered on a cBot Cluster Generation System using a TruSeq PE Cluster Kit v3-cBot-HS (Illumina). We sequenced the cDNA libraries on an Illumina HiSeq 2000 platform and paired-end reads were generated, with each read length of 50 bp.

### De novo assembly and gene functional annotation

Raw data (raw reads) were firstly processed with in-house Perl scripts (Additional file 3), and clean data (clean reads) were then obtained by removing reads containing adapters, reads containing poly-N and reads of low quality (reads with more than 5% unknown nucleotides or reads of less than 13 bp) from the raw data. The number of raw reads, clean reads and Q20 and Q30 values were calculated at the same time. All subsequent analyses were based on the clean data of high quality. Due to the absence of reference sequences for *R. omeimontis*, high-quality clean data were de novo assembled into transcripts using Trinity software with min_kmer_cov set to 2 by default and all other parameters set to the default [29]. Unigenes were selected from the longest transcript copy of each gene cluster to avoid redundant transcripts [84]. All unigenes were functionally annotated against the following databases: the NCBI non-redundant protein sequences (NR) database, the Protein family database (Pfam) database (http://pfam.xfam.org/), the manually annotated and reviewed protein sequence database (Swiss-Prot) database, the Clusters of Orthologous Groups of Proteins (COG) database, the Pathway Annotation of the Kyoto Encyclopedia of Genes and Genomes (KEGG) database and the Gene Ontology (GO) database. GO annotation was performed using the Blast2GO method [85, 86].

### Differential expression analyses

Gene expression levels were quantified by RSEM for each sample [28]. First, clean data were mapped back onto the assembled transcriptome. Then, the read count for each gene was obtained from the mapping results. The gene expression level was estimated by calculating the fragments per kb per million reads (FPKM). Differential expression analysis between the two periods of foam nest construction was performed using the DESeq R package [87], and the resulting *p* values were adjusted using Benjamini and Hochberg’s approach for controlling the false discovery rate (FDR) [88]. The genes with an adjusted *p* value < 0.05 and |log2 (fold change) | > 1 were designated as significant differentially expressed genes (DEGs).

GO enrichment of the DEGs was performed with the goseq R packages based on Wallenius’ non-central hyper-geometric distribution [86]. The KEGG database is a database resource for understanding high-level functions and utilities of biological systems, especially large-scale molecular datasets generated by genome sequencing and other high-throughput experimental technologies (http://www.genome.jp/kegg/) [89]. KEGG enrichment of the DEGs was applied using KOBAS software and the ‘fdr’ parameter set to ‘BH’ [90].

### qRT-PCR validation

To validate the accuracy of our transcriptome data, we selected 14 genes from the significantly differentially expressed genes and performed qRT-PCR using a Bio-Rad CFX96™ Real-Time System. Total RNA was extracted from the oviducts for the two periods using TRIzol reagent. The PCRs were conducted using SYBR Green I dye with a 20-μl-total volume mixture containing 2 μl of cDNA as the template. The expression levels of the genes were normalized using the housekeeping gene GAPDH as an endogenous control, and the relative gene expression was calculated by the 2^-ΔΔCT^ method. All experiments were performed with three biological replicates, and the data were analysed by one-way analysis of variance (ANOVA). The primers used in this study were designed with Primer Express 3.0 software, and additional information about the primers is provided (Additional file 1: Table S4).

## Additional files

Additional file 1: **Table S1-S9. Table S1:** The statistics and quality of reads for the six samples of the *Rhacophorus omeimontis* transcriptome. **Table S2:** The upregulated differentially expressed genes in the oviduct of *Rhacophorus omeimontis*. **Table S3:** The downregulated differentially expressed genes in the oviduct of *Rhacophorus omeimontis.* **Table S4:** The primers used for quantitative RT-PCR analysis in this study. **Table S5:** GO enrichment of the upregualted DEGs. **Table S6:** GO enrichment of the downregualted DEGs. **Table S7:** KEGG enrichment of the upregualted DEGs. **Table S8:** KEGG enrichment of the downregualted DEGs. **Table S9:** The annotation information of all unigenes. (XLSX, 14,044 kb) (XLSX 14036 kb)

Additional file 2: **Figure S1-S2. Figure S1.** The unigene length distribution of the *Rhacophorus omeimontis* transcriptome. **Figure S2.** Significant enrichment analysis of GO terms in the upregulated differentially expressed genes. (DOCX, 119 kb) (DOCX 118 kb)

Additional file 3: The Perl scripts for transcriptome analysis in this study. (ZIP 5 kb)

## Data Availability

The dataset supporting the conclusions of this study is included within the article and its additional files. The raw data of this study has been deposited in the NCBI Sequence Read Archive (SRA) database under the accession PRJNA529065. The assembled sequence has been deposited at DDBJ/EMBL/GenBank under the accession GHMZ00000000. The version described in this paper is the first version, GHMZ01000000.

## Abbreviations

APOC-1: Apolipoprotein C-I-like gene
C7: Complement component 7
cDNA: DNA complementary to RNA
CYP11A1: Cytochrome P450 family 11 subfamily A polypeptide 1
DEGs: Differentially expressed genes
FDR: False discovery rate adjusted *p* value
GO: Gene Ontology database
KEGG: Kyoto Encyclopedia of Genes and Genomes database
KOG/COG: Clusters of Orthologous Groups of proteins database
MHC: Major histocompatibility complex
NR: non-redundant protein sequences database
PARP14: Poly [ADP-ribose] polymerase 14 gene
Pfam: Protein family database
qRT-PCR: Quantitative reverse transcription real-time PCR
REG: Regenerating islet-derived protein gene
Swiss-Prot: the manually annotated and reviewed protein sequence database
VOM1: Vitelline membrane outer layer protein 1 homolog precursor
