# Supplementary material for: Transcriptomic analysis of differentially expressed genes in the oviduct of Rhacophorus omeimontis provides insights into foam nest construction
Source: BMC Genomics. 2019 Jul 8;20:562. doi: 10.1186/s12864-019-5931-7 (PMC6615284; doi:10.1186/s12864-019-5931-7)
Supplement: Supplementary file 2 — Figure S1-S2. Figure S1. The unigene length distribution of the Rhacophorus omeimontis transcriptome. Figure S2. Significant enrichment analysis of GO terms in the upregulated differentially expressed genes. (DOCX, 119 kb) (DOCX 118 kb) [file 12864_2019_5931_MOESM2_ESM.docx]

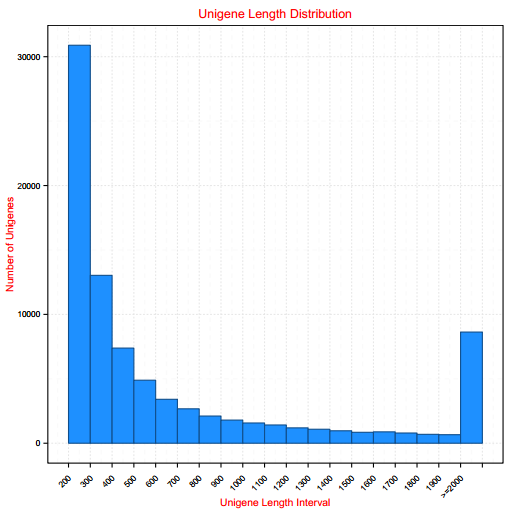


Figure S1 The unigene length distribution of *Rhacophorus omeimontis* transcriptome.


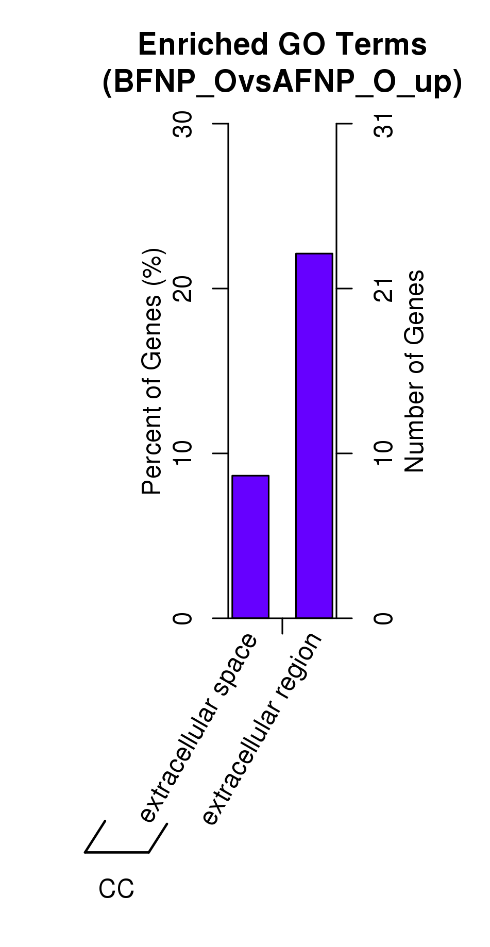


Figure S2 The significant enrichment analysis of GO term in the up-regulated differentially expressed genes.
